# Automatic vessel plate number recognition for surface unmanned vehicles with marine applications

**DOI:** 10.3389/fnbot.2023.1131392

**Published:** 2023-04-20

**Authors:** Renran Zhang, Lei Zhang, Yumin Su, Qingze Yu, Gaoyi Bai

**Affiliations:** Department of Science and Technology on Underwater Vehicle Laboratory, Harbin Engineering University, Harbin, China

**Keywords:** YOLOv5, vessel plate number, unmanned surface vehicles (USVs), real-time recognition, K-means++ algorithm

## Abstract

In the practical application scenarios of USVs, it is necessary to identify a vessel in order to accomplish tasks. Considering the sensors equipped on the USV, visible images provide the fastest and most efficient way of determining the hull number. The current studies divide the task of recognizing vessel plate number into two independent subtasks: text localization in the image and its recognition. Then, researchers are focusing on improving the accuracy of localization and recognition separately. However, these methods cannot be directly applied to USVs due to the difference between these two application scenarios. In addition, as the two independent models are serial, there will be inevitable propagation of error between them, as well as an increase in time costs, resulting in a less satisfactory performance. In view of the above, we proposed a method based on object detection model for recognizing vessel plate number in complicated sea environments applied to USVs. The accuracy and stability of model have been promoted by recursive gated convolution structure, decoupled head, reconstructing loss function, and redesigning the sizes of anchor boxes. To facilitate this research, a vessel plate number dataset is established in this paper. Furthermore, we conducted a experiment utilizing a USV platform in the South China Sea. Compared with the original YOLOv5, the mAP (mean Average Precision) value of proposed method is increased by 6.23%. The method is employed on the “Tian Xing” USV platform and the experiment results indicates both the ship and vessel plate number can be recognized in real-time. In both the civilian and military sectors, this has a great deal of significance.

## 1. Introduction

Unmanned Surface Vehicles (USVs) have received considerable attention due to their high working efficiency and strong adaptability. In recent years, USVs have been widely applied in marine safety filed. They can accomplish the missions such as emergency rescue and maintaining safety in waterway traffic navigation (Yang et al., 2018; He et al., 2022). As the unique identification of a ship, the vessel plate number plays an indispensable role in the process of the USVs carrying out the missions above. In practice, identifying a ship through the vessel plate number can provide the basic information of the target vessels for USVs, which contributes to responding to an emergency (Dobref et al., 2018). In general, the methods for identifying a ship depending on specific facilities such as Automatic Identification System (AIS), Long-Range Identification and Tracking (LRIT) (Harati-Mokhtari et al., 2007; García-Silveira et al., 2022; Pouyaei et al., 2022). However, due to volume limitations and stealth requirements, the USVs will not carry or open the above equipment in most cases. Thus, the hull identification number detection based on visual images become more critical to the mission's success.

In recent years, many researchers have contributed and provided different methods to identify ship. Zhang et al. (2018) focused on common ships at the marine port of Dongying that have conventional, large, and distinct IMO hull markings, often locally accompanied by Chinese characters. They propose the FCNPR (Fully Convolutional Network based Plate Recognition) approach which use a SSD network to locate ship and complete the text line detection on ship with a full connection neural network. Cropped image containing text is fed into a pretrained classification model which is integrated with AIS information to obtain the recognition result. The shortcoming of this method is that it cannot meet the real-time requirement. To improve the speed of ship identification, Huang et al. (2018) present an end-to-end solution for vessel plate number detection and recognition simultaneously. The network combines a CNN network for detection of vessel plate number region and LSTM with CTC for recognition of vessel plate. But these two researches concerned only large ships with standardized hull markings, with no consideration of that the font and size that are not standardized. To cover this drawback, Wawrzyniak et al. (2022) propose a method based on a combination of different text localization methods and additional processing and comparison of various character strings with existing ship identification data registers. The experiments illustrate that this method can recognize a wide range of vessels of many types using different hull marking rules. In these studies, the task of recognizing vessel plate number is divided into two independent subtasks, text localization in the image and its recognition. Although researchers endeavor to separately improve the accuracy of localization and recognition, these methods cannot be directly applied to USVs because of the higher real-time need in its application scenarios. Since the text recognition model is aimed at typical flat-view and close-range shooting scenarios which cannot perform well in the practical application scenarios for USVs. Furthermore, due to the series connection between the two models, the propagation of error between them as well as an increase in time costs will not be ignored.

As the vessel plate number is generally composed of numbers, it is feasible to identify a single number based on the target detection model and then complete the vessel plate number identification. And the disadvantages caused by using two serial models can be avoided. Therefore, this paper proposes a target detection method to complete the task of vessel plate number recognition.

At present, most target detection methods are based on anchor boxes, and these detection methods are divided into single stage and two stages. The two-stage method improves the accuracy through the regional recommendation network, and then forecasts based on the anchor. The representative of two-stage method is R-CNN series network (He et al., 2017). These methods have higher accuracy, but the detection speed is not satisfactory. The single-stage method can predict directly through the anchor, although the accuracy has decreased, it has faster detection speed. Released by Redmon et al. (2016) in 2015, YOLOv1 is the first work of one stage detection. Redmon and Farhadi proposed the YOLOv2 (Redmon and Farhadi, 2017) algorithm in 2017. The author proposes to improve YOLOv1 algorithm from three aspects of more accurate, faster and more recognition, in which the recognition of more objects is expanded to detect 9000 different objects. In 2018, Redmon and Farhadi proposed YOLOv3 (Redmon and Farhadi, 2018), which is an improvement made previously. The biggest improvement features include the use of residual model Darknet-53, and the use of FPN architecture to achieve multi-scale detection. On the original basis, YOLOv4 (Bochkovskiy et al., 2020) and YOLOv5 optimize the backbone network, network training, activation function, loss function, etc.

Motivated by the above observation, this paper developed a method based on YOLOv5 for recognizing vessel plate number in complicated sea environments applied to USVs. The proposed method for recognizing vessel plate numbers differs significantly from previous methods in the following respects:

- As the vessel plate number is smaller than that of ship, the feature of the vessel plate number would be lost during the feature extraction process. In view of this shortcoming, the recursive gated convolution structure is introduced to perform high-order spatial interactions with gated convolutions and recursive designs, which could improve the model's capacity for extracting features.
- The loss function is reconstructed considering the angle of the vector between the desired regression to improve both the convergence speed and the accuracy of the inference.
- The classification task focuses on the salient region features while the regression task focuses on the edge features. Through shared weights, the coupled detection head performs classification and location simultaneously. However, the conflict between the two tasks reduces accuracy. In this paper, the decoupled detection head is utilized to avoid the conflict between classification and regression.
- The proposed method is an anchor box-based detection method and the anchor boxes which are aligned with the dataset distribution will yield more accurate results. Based on the K-means++ algorithm cluster algorithm, the sizes of anchor boxes are redesigned in this paper.

The main contributions of this paper are summarized as follows. Traditional methods based on localization and recognition models suffer from the high complexity and error propagation between these two models. Therefore, we perform both detection and classification based on the target detection model, deprecating the text recognition stage. The native YOLOv5 performs well in object detection with a high degree of accuracy, while the similarity between single numbers limits its detection and classification capabilities. To guarantee high-quality detection results, the feature extraction part, the loss function and the head part of the model are reconstructed. Different from the previous literature, an innovative one-stage model structure is proposed and designed in this work to complete the ship plate number mission for USVs. The proposed model is implemented on the “Tian Xing” USV platform for testing performance, and the results of the experiments indicate that it performs better than the original model.

The structure of this article is organized as follows: The proposed methods are explained in detail in Section 2. Section 3 presents the experimental results and a discussion. The paper concludes with Section 4.

## 2. Methods

**Figure 1:**
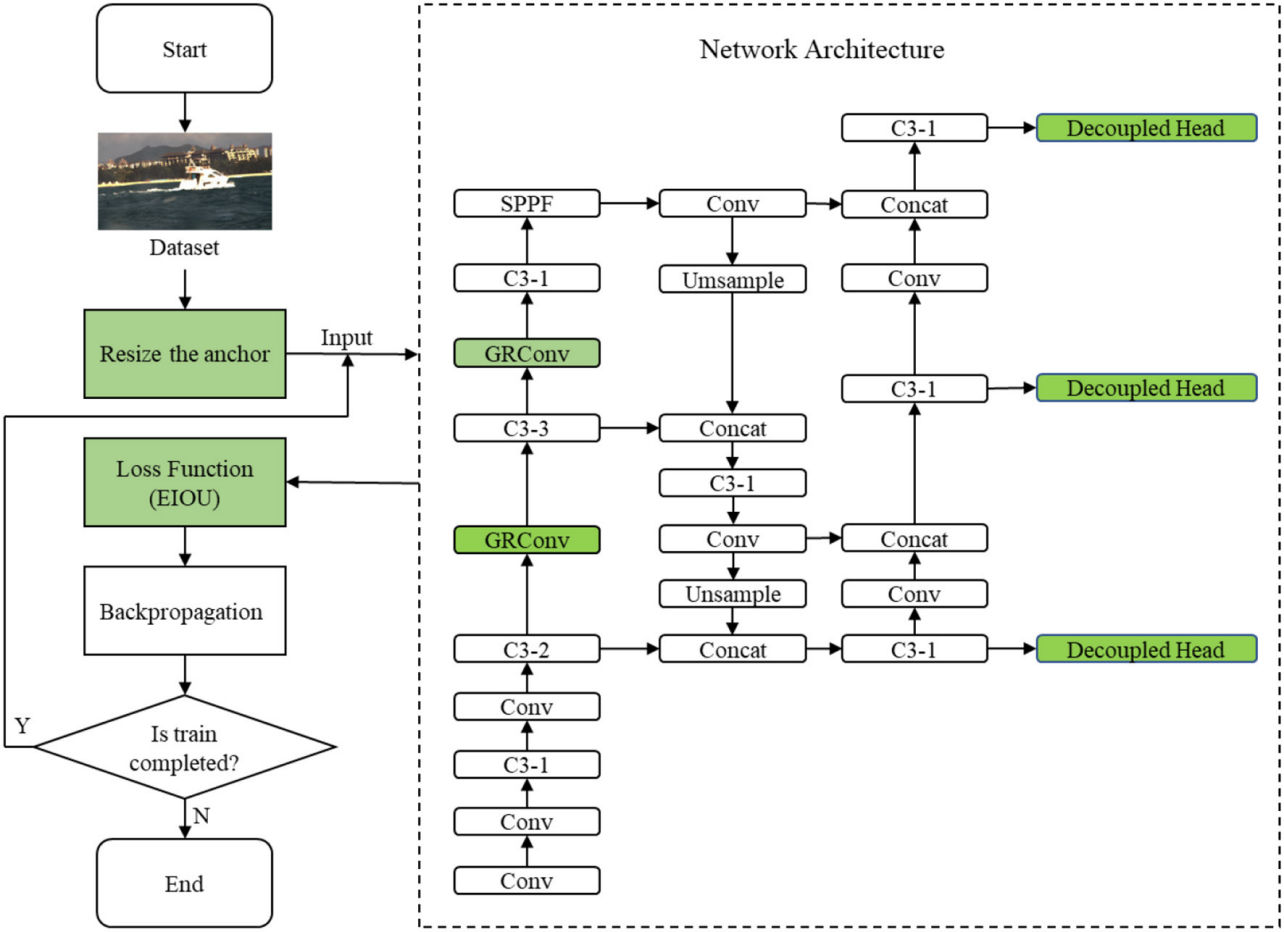
Workflow diagram of the proposed network.

For electro-optical sensors employed on USVs, the vessel plate number occupies fewer pixels in images leading loss of detail, that further increases the difficulty of regression for detection and classification simultaneously. To cover this drawback, we propose to perform detection on different scales feature layers to improve the performance on vessel plate number recognition. To be specific, we will introduce recursive gated convolution to perform high-order spatial interactions without extra computation in Section 2.1. In addition, the decoupled head strategy will be exploited in Section 2.2 to improve the accuracy of detection. The proposed decouple head for classification and regression can resolve the coupled problem caused by parameter sharing in detect layer. In Section 2.3, the reconstruction of loss function improves the convergence speed and precision by considering the angle of the vector between the desired regression. In Section 2.4, the size of anchor is redesigned to improve the accuracy. Combing the above strategies, the enhanced YOLOv5 has the capacity to detect ship and recognize the vessel plate number simultaneously with more accurate results and faster speed meeting the requirements of application on USVs. The work flow of our detection model in training is shown in Figure 1.

### 2.1. The recursive gated convolution block

The Convolutional Neural Networks (CNNs) have driven remarkable progress in detection model based on deep learning. The main advantage of CNN compared to its predecessors is that it automatically detects the important features without human supervision. In the CNN structure, each neuron is connected only to a small chunk of the input, meanwhile, all the neurons have the same connection weights. These two operations can make CNN obtain the detail features with less computational costs. However, the strong detail capture capability of CNN makes it be limited in capturing global features. Inspired by previous work (Rao et al., 2022), we propose to introduce the recursive gated convolution to perform high-order spatial interactions. Let *x* ∈ *R*^*HW* × *C*^ be the input feature, the output is shown as followed:


(1) $$\begin{array}{l} \left[{{\text{p}}_{0}}^{HW\times C},{{\text{q}}_{0}}^{HW\times C}\right]=\phi_{\text{in}}\left(\text{x}\right)\in {\mathbb{R}}^{HW\times 2C},\\ {\text{p}}_{1}=f\left({\text{q}}_{0}\right)⊙\;{\text{p}}_{0}\in {\mathbb{R}}^{HW\times C},\text{y}=\phi_{\text{out}}\left({\text{p}}_{1}\right)\in {\mathbb{R}}^{HW\times C} \end{array}$$

Where *ϕ*_in_, *ϕ*_out_ represent linear convolution operation to perform channel mixing, and indicates a depth-wise convolution.

The formulation Equation 1 introduce the 1-order interaction among the features ${{\text{p}}_{0}}^{\left(i\right)}$ and ${{\text{q}}_{0}}^{\left(i\right)}$ through the element-wise multiplication once. Similarly, the n-order form is formulated as:


(2) $$\begin{array}{l} \left[{p_{0}}^{HW\times C_{0}},{q_{0}}^{HW\times C_{0}},...,{q_{n-1}}^{HW\times C_{n-1}}\right]=\\ \;\phi_{\text{in}}\left(x\right)\in {\mathbb{R}}^{HW\times \left(C_{0}+\sum_{0\leq k\leq n-1}C_{k}\right)} \end{array}$$

(3) $$\begin{array}{l} P_{k+1}=f_{k}\left(q_{k}\right)⊙g_{k}\left(p_{k}\right)/\alpha ,k=0,1,...,n-1 \end{array}$$

Where the output is scaled by 1/α, and *g*_*k*_ are used to match the dimension in different orders in Equation 4.


(4) $$g_{k}=\{\begin{array}{cc} Indentity, & k=0,\\ Linear\left(C_{k-1},C_{k}\right), & 1\leq k\leq n-1 \end{array}$$

From the recurise formular Equation 3, we can see that recursive gated convolution block achieves n-order spatial interactions. And the channel dimension in each order is set as the Equation 5 to avoid computational overhead.


(5) $$\begin{array}{l} C_{k}=\frac{C}{2^{n-k-1}},\text{0}\leq k\leq n-1 \end{array}$$

Where the *C* indicates the number of channels.

**Figure 2:**
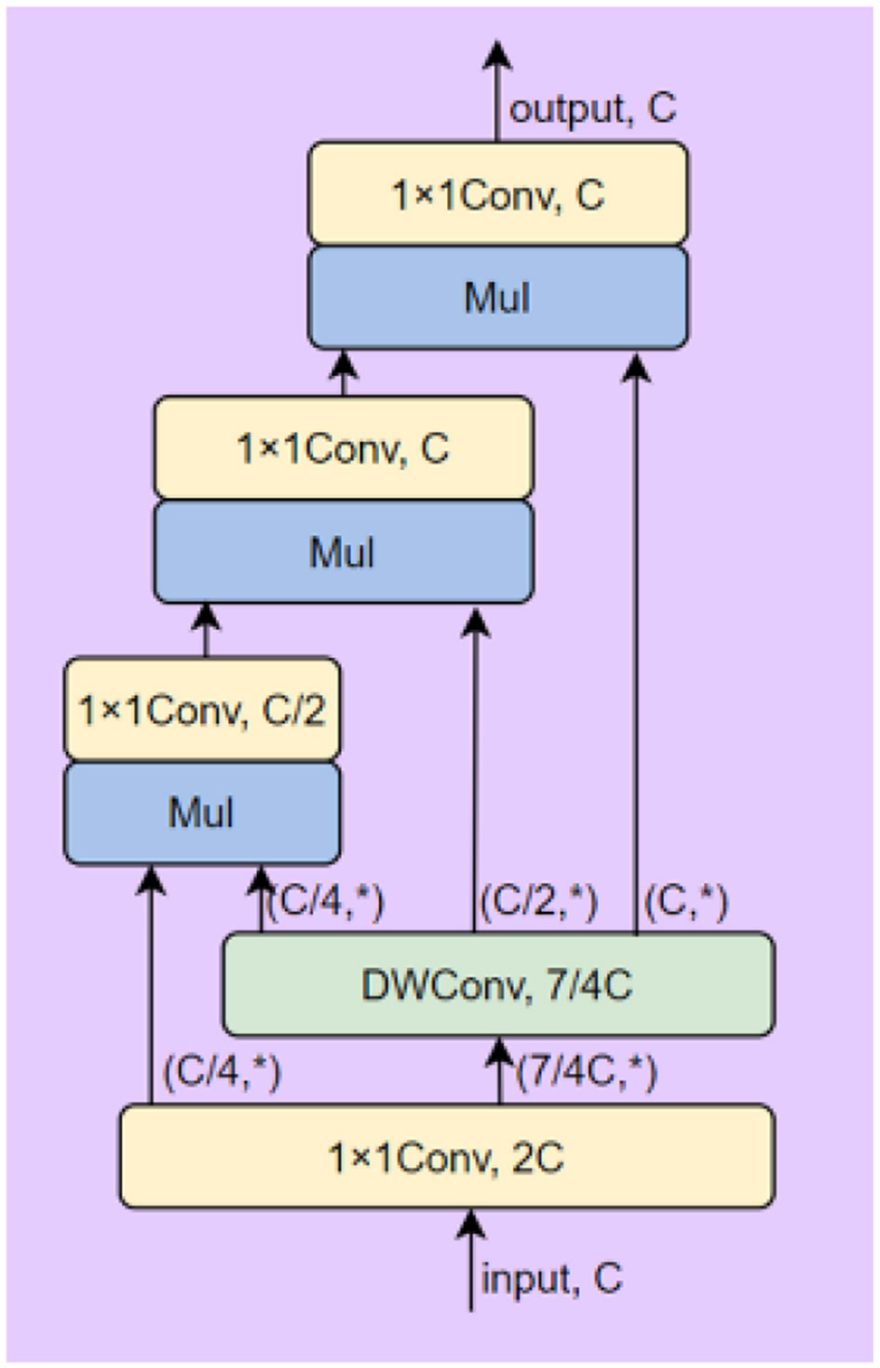
The structure of the recursive gated convolution block.

This block can perform high-order spatial interactions to improve the learning and capacity of the neural network without extra computation. The details of model are show in Figure 2.

### 2.2. Decoupled head

In original YOLOv5, the classification and regression are completed simultaneously in detect layer with the same input feature map. However, there is conflict caused by spatial misalignment between classification and boundary regression, which may harm the performance of detection model (Ge et al., 2021). To be specific, a detector can hardly get a perfect trade-off result if accomplishing classification and regression from a same spatial point/anchor. Motivated by (Revisiting the Sibling Head in Object Detector), the decoupled head method is introduced which decouples these two tasks from spatial dimension by two disentangled proposals.

**Figure 3:**
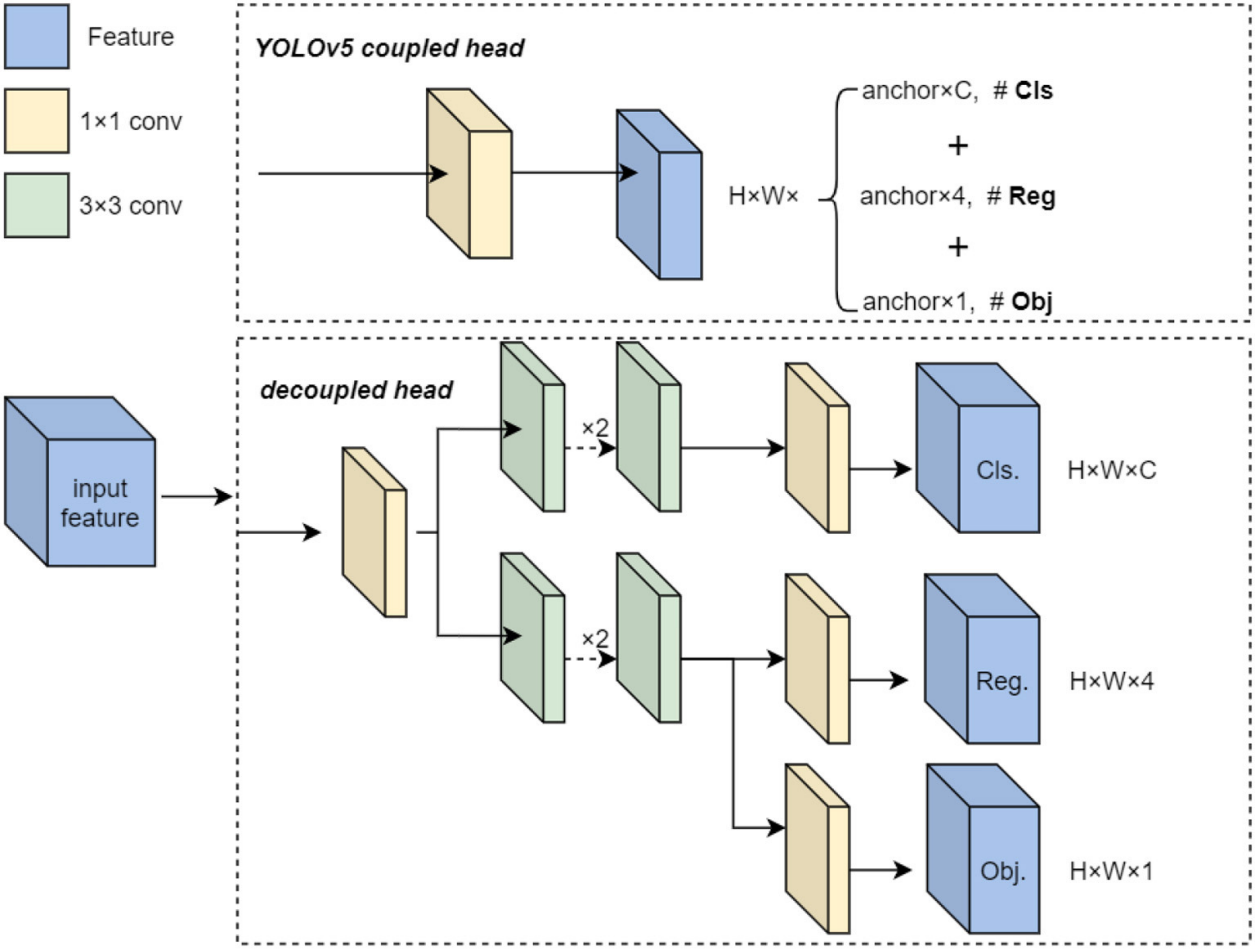
The architecture of decoupled head.

According to the observation above, the decoupled head is utilized to predict the class and localization instead of original detect head in YOLOv5. Different from the method in (Rethinking Classification and Localization for Object Detection), we propose a lite decoupled head method without fully connected layers to meet the requirement of real-time detection for USVs. The decouple head splits the classification and bounding box regression into two convolution heads, which have the identical structure with independent parameters. The details of proposed structure are shown in Figure 3.

As show in the Figure 3, the num of channels is firstly adjusted to 256 by convolution layer with 1x1 kernel. Here the * indicates the width and height stay the same as the input. Then the intermediate result is fed into the predict part constructed with two parallel branches, whereas one branch for classification and the other for regression. This operation can resolve the coupling problem exists between two tasks, which effectively improve the performance of detection model.

### 2.3. Reconstruction of bounding box loss

In the training phrase, the parameters of the model are updated according the result of loss function. The loss of YOLOv5 is calculated based on objection score, class probability score, and bounding box regression score, whereas the Binary Cross the bounding box regression score is calculated by CIOU. The CIOU expression is as follows.


(6) $$\begin{array}{l} \begin{array}{c} CIOU=IOU-\frac{\rho^{2}\left(\text{b}\text{,}{\text{b}}^{gt}\right)}{c^{2}}-\beta v,\\ v=\frac{4}{\pi^{2}}{\left(\text{arctan}\frac{w^{gt}}{h^{gt}}-\text{arctan}\frac{w}{h}\right)}^{2} \end{array} \end{array}$$

Where indicates the width and height of box respectively, ρ^2^(**b**, **b**^*gt*^) represents the Euler distance square of the center of the prediction box and the truth box, and *c*^2^ represents the diagonal distance square of the maximum circumscribed matrix between the prediction box and the truth box. β is the aspect ratio influence factor, and *v* represents the penalty items of the prediction box and the truth box. It can be seen from the formula that CIOU takes into account the center distance, area overlap, and aspect ratio of the prediction box and the truth box. Compared with ordinary IOU, it can more effectively reflect the similarity of the target box. Therefore, the loss design method based on CIOU can make the model training converge faster.

However, CIOU only takes the width height ratio as the influence factor, and does not explicitly consider the width height value (Zheng et al., 2020). For this reason, EIOU takes the length width influence factor as the penalty item, rather than the length width ratio (Yang et al., 2021). The formula is as follows,


(7) $$\begin{array}{l} EIOU=IOU-\frac{\rho^{2}\left(b,b^{gt}\right)}{c^{2}}-\frac{\rho^{2}\left(w\text{,}w^{gt}\right)}{{c_{w}}^{2}}-\frac{\rho^{2}\left(h\text{,}h^{gt}\right)}{{c_{h}}^{2}} \end{array}$$

Where, $\frac{\rho^{2}\left(w\text{,}w^{gt}\right)}{{c_{w}}^{2}}$ and $\frac{\rho^{2}\left(h\text{,}h^{gt}\right)}{{c_{h}}^{2}}$ represent width influence factor and length influence factor respectively. Because EIOU directly uses the length and width of the target box as the penalty term, it will theoretically bring faster convergence speed to the model training.

### 2.4. Redesigning the sizes of anchor boxes

The YOLOv5 is a model based on anchor, so the prior design of anchor size is very important. The anchor size of YOLOv5 is set according to the COCO dataset to obtain different aspect ratios of large, medium and small targets. However, there is a significant difference in size between ships and vessel plate number. There should be a recalculation of anchor size. After analysis, we simply use the K-means algorithm to regress, and the results obtained are not necessarily optimal, because the random initial values of the K-means algorithm have a greater impact on the results, and the robustness of the algorithm is poor.

Based on the above considerations, we adopted the K-means++ algorithm, hoping to obtain a more reasonable anchor size prior. The K-means++ algorithm process is as follows:

a). Choose one center uniformly at random among the data points.
b). For each data point x not chosen yet, compute*D*(*x*), the distance between x and the nearest center that has already been chosen.
c). Choose one new data point at random as a new center, using a weighted probability distribution where a point x is chosen with probability proportional to.
d). Repeat Steps 2 and 3 until k centers have been chosen.
e). Now that the initial centers have been chosen, proceed using standard k-means clustering.

**Figure 4:**
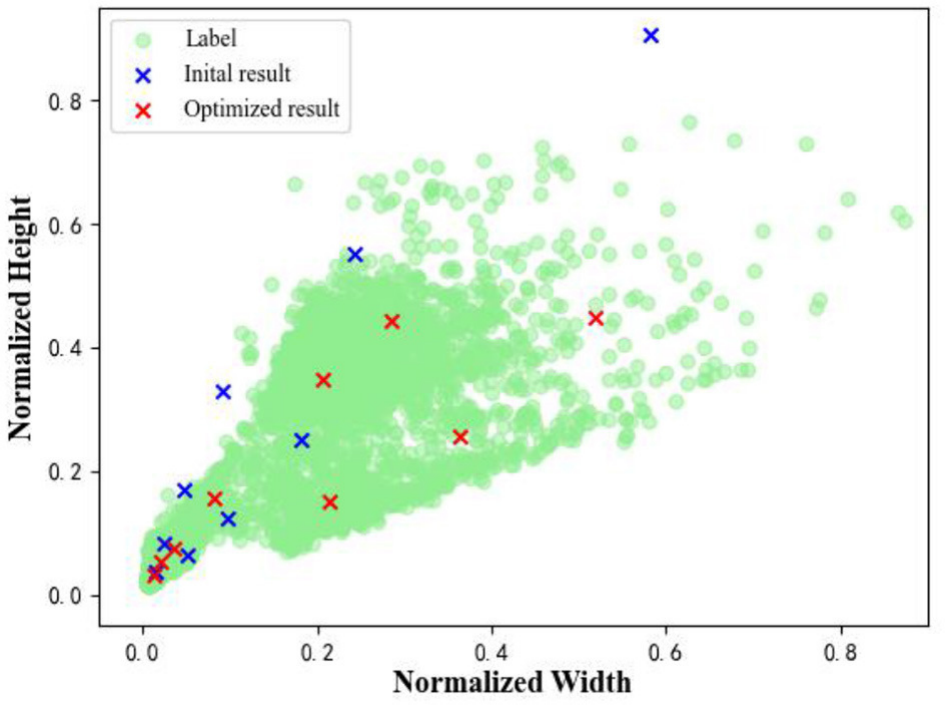
The comparison of anchor size.

This method was tested on our own dataset and the results are shown in Figure 4. The default anchor size (blue) is (10, 16, 33, 30, 62, 59, 116, 156, 373) and the optimized results (red) is (10, 16, 33, 30, 62, 59, 116, 156, 373). It can be seen that the optimized anchor is more consistent with the real data distribution, which can improve the performance of the model.

## 3. Experiment and result analysis

To evaluate the performance of the proposed method, we conducted detection experiments on the computer carried with the USVs. In particular, all experiments are conducted on a computer with Intel(R) Core(TM) i5-9600K@3.7GHz CPU and NVIDIA GeForce RTX2080Ti GPU. The code was written in Python using the Pytorch software library and executed under Ubuntu 20.04.

### 3.1. Dataset description

**Figure 5:**
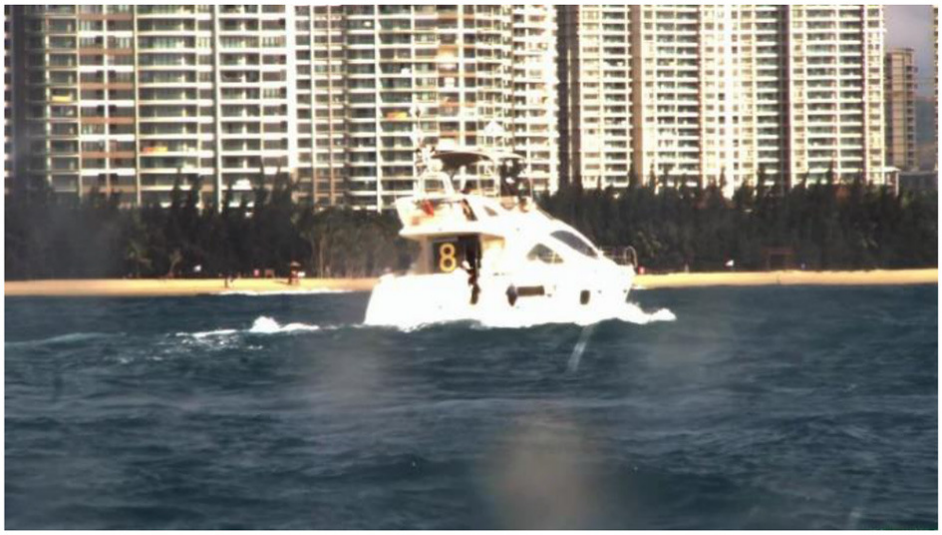
The target boat.

The deep neural network are trained and verified based on a dataset, however, there is no relevant public dataset. In this paper, we propose to establish a vessel plate number dataset in USVs perspective. All the images are obtained from the electro-optical sensor carried by USVs. To increase the diversity of scenes, the number and symbol are displayed on the LED board (1.5 m × 1.5 m) carried on the target boat (10 m) and the content in the LED changes periodically as shown in Figure 5.

**Figure 6:**
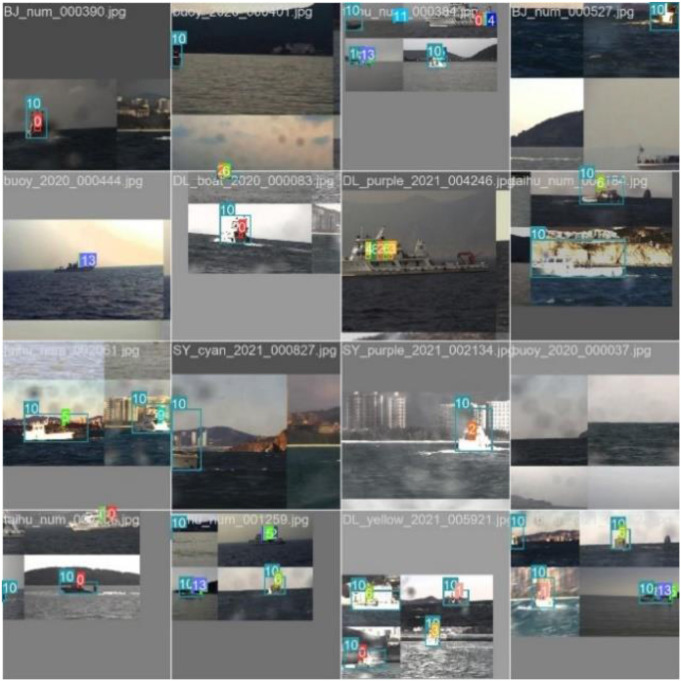
The samples of dataset.

The dataset contains 5011 images and covers 16 types of objects, i.e., ship, buoy, single number, symbols(star, rectangle, triangle). The dataset are divided into training set and verification set according to ratio of 8:2. To further improve ship detection results, we propose to exploit the data augmentation methods, e.g., horizontal flipping, random translation, and mosaic augmentation, etc., to enlarge the original training dataset, shown in Figure 6.

### 3.2. Evaluation criteria

To quantitatively evaluate the detection results, the P(Precision), R(Recall), mAP are utilized in this paper. In particular, the P is the ratio of the number of true positives to the total number of positive predictions. The R is the ratio of the number of true positives to the total number of actual (relevant) objects. The mAP computes the average precision value for recall value which indicates the detection robustness and accuracy. The method used to calculate the mAP is the following formula:


(8) $$\begin{array}{l} \text{mAP=}\frac{\text{1}}{\text{N}}\overset{\text{N}}{\underset{\text{n=1}}{\sum }}\text{A}{\text{P}}_{\text{n}} \end{array}$$

Where the average precision score AP_n_ is calculated for N data folds.

In this paper, the mAP@0.5:0.95 is adopted as the mAP criteria which represent the average mAP on different IOU thresholds (from 0.5 to 0.95, in steps of 0.05).

### 3.3. Model training

**Figure 7:**
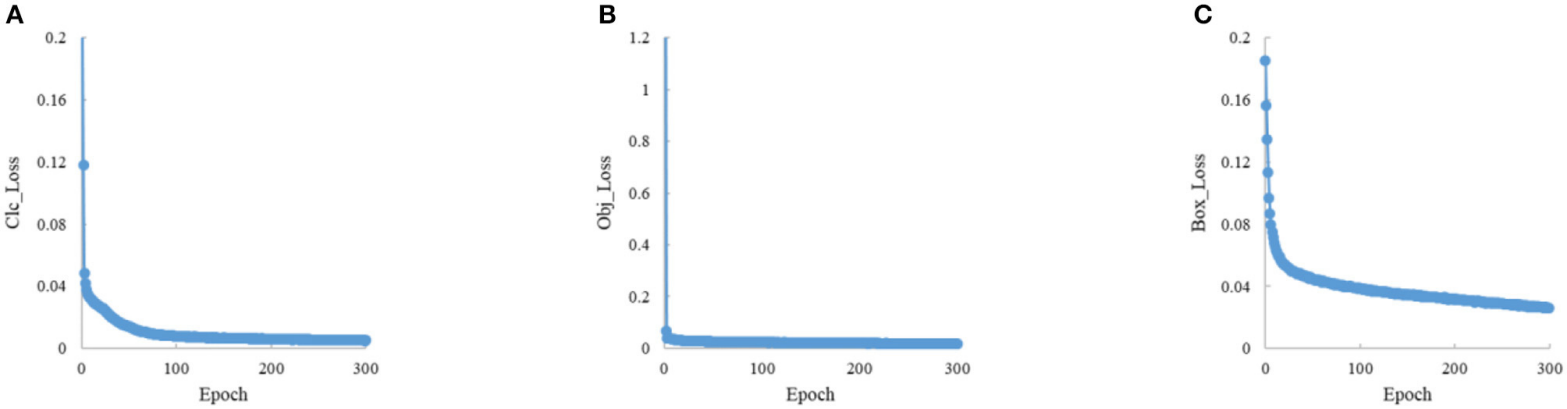
The training loss convergence curve. **(A)** The loss of class. **(B)** The loss of object. **(C)** The loss of box.

**Figure 8:**
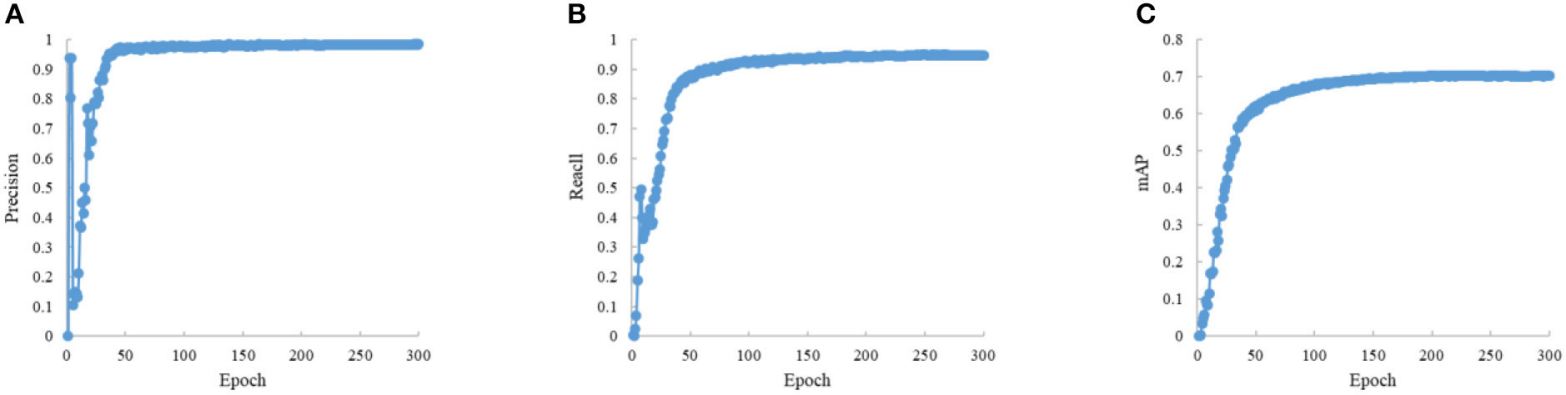
The metric convergence curve. **(A)** The curve of metric P. **(B)** The curve of metric R. **(C)** The curve of metric mAP.

In the experiments, the input image size is 600 × 600, the training epoch is 300, the batch size is 16, the optimizer is SGD, and the initial learning rate is 0.01. To ensure the stability of convergence, the cosine annealing strategy is used to dynamically adjust the learning rate during training. The results are shown in Figures 7, 8.

The train loss convergence curves are shown in Figure 7, containing the bounding box loss, confidence loss and classification loss. The loss function tends to convergence within the first 100 epochs, which indicates that the proposed method is stable and fast in convergence. The Figure 8 indicates the proposed model performance well in vessel plate number recognition task.

### 3.4. Ablation experiments

As discussed in Section 2, the vessel plate number recognition model is proposed by taking into consideration several modules, e.g., recursive gated convolutions (RGConv), decoupled head (DH), EIOU, adaptive anchor size (AAS). Therefore, ablation experiments will be performed to determine which one improves detection performance more effectively. The detailed description of the numerical experiments can be found in Table 1.

**Table 1:** The comparisons result of ablation experiments.

**Methods**	**RGConv**	**AAS**	**DH**	**EIOU**	**mAP (%)**
Original	x	x	x	x	64.12
Proposed	✓	x	x	x	67.51
	✓	✓	x	x	68.03
	✓	✓	✓	x	70.38
	✓	✓	✓	✓	70.35

It is shown that the accuracy is lowest for the original YOLOv5. The introduction of RGConv and DH have the potential to enhance the accuracy of detection. It seems that the recognition accuracy, brought by EIOU, is not obvious. However, it is found in the training phase that the method with EIOU converges more steadily that the original YOLOv5. When compared to the original YOLOv5, the proposed method improves the mAP by 6.23%. As a consequence, the introduction of RGConv, AAS, DH, EIOU can bring positive effects on improved vessel plate number recognition results.

### 3.5. The experiment on USV

**Figure 9:**
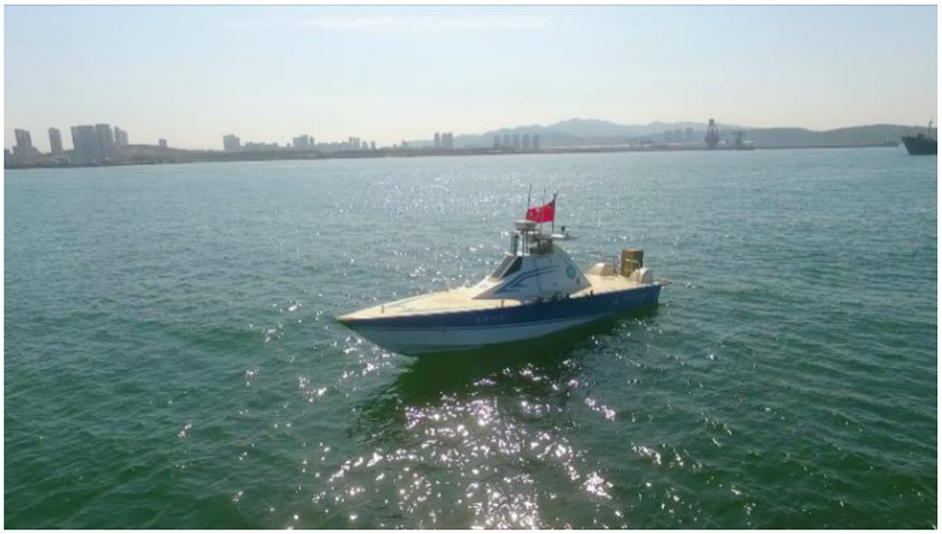
“Tian Xing” USV.

**Figure 10:**
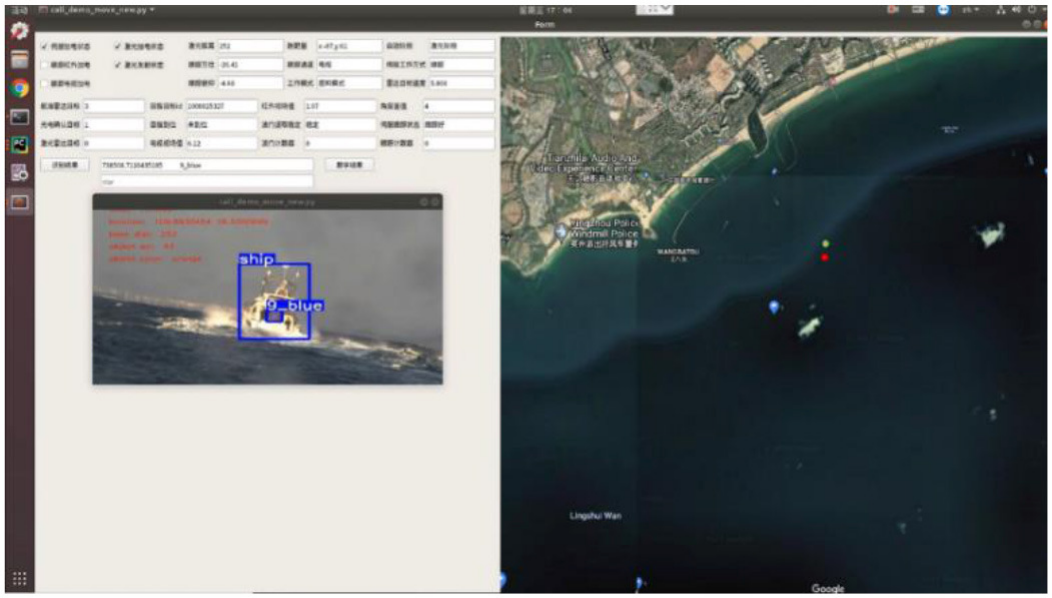
The software interface for perception system in USV.

**Figure 11:**
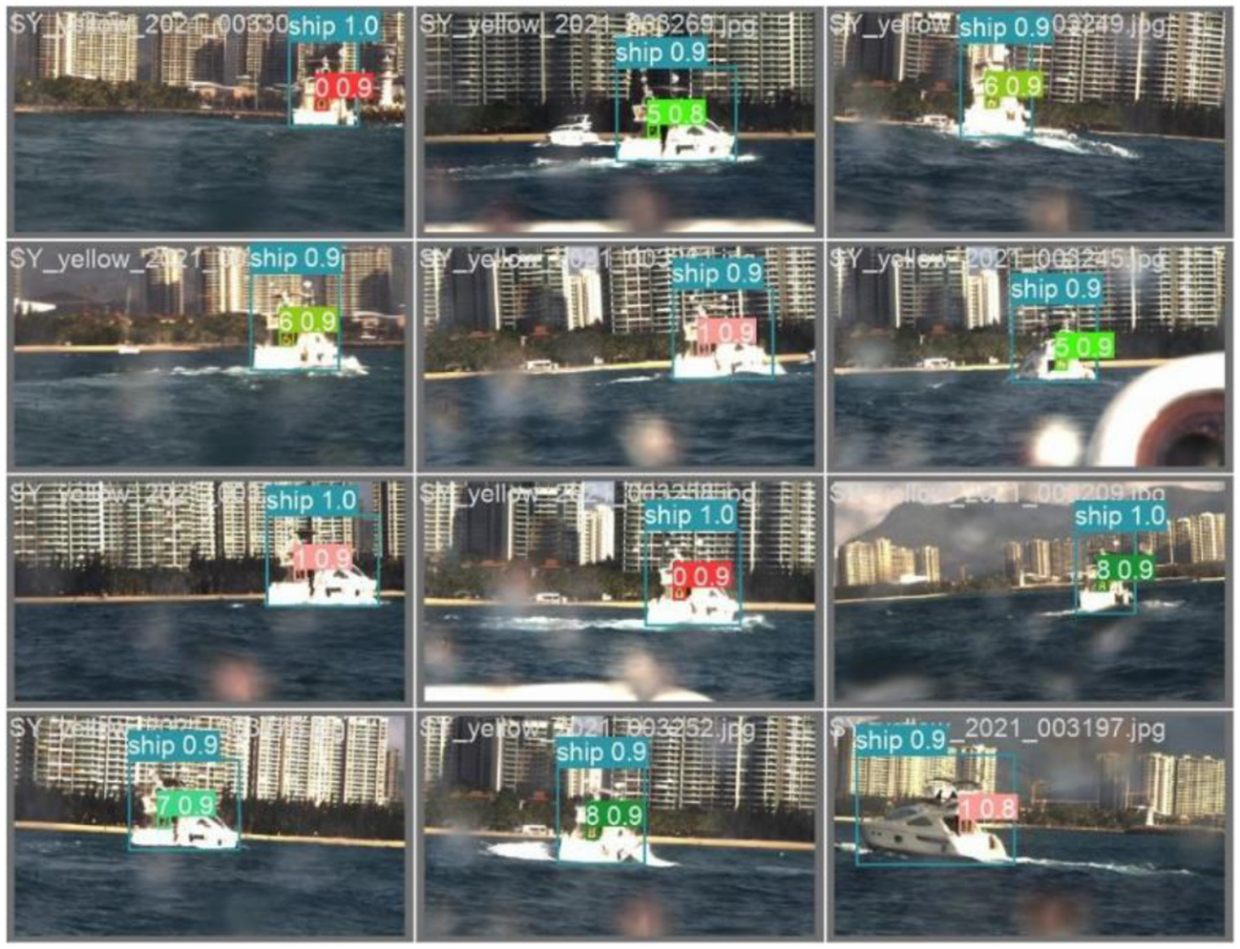
The samples of recognition results.

The method proposed in this paper was tested in the South China Sea in order to verify its practicability. The “Tian Xing” USV platform in the experiment can be seen in the Figure 9. The object boat is equipped with LED board that display the hull number. The visualization results are shown in Figures 10, 11. The Figure 10 represents the software interface that displays environment perception information for the USV. It can be found that the hull number of the target boat can be correctly identified by the proposed method while guaranteeing real-time detection results.

## 4. Conclusion

In the practical application tasks of USVs, it is necessary to identify a vessel through its plate number. In this work, we proposed a method based on object detection model for recognizing vessel plate number in complicated sea environments applied to USVs. The accuracy and stability of model have been promoted by recursive gated convolution structure, decoupled head, reconstructing loss function, and redesigning the sizes of anchor boxes. To facilitate this research, a vessel plate number dataset is established in this paper. Furthermore, we conducted a field experiment with the “Tian Xing” platform in the South China Sea. Compared with the original YOLOv5, the proposed method could real-timely recognize both the ship and its plate number with higher accuracy. In both the civilian and military sectors, this has a great deal of significance.

Although the proposed method has achieved good results in the recognition of vessel plate numbers, it still has room for improvement. In addition, this paper does not consider the impact of ocean climate on recognition accuracy. Changes in climate often result in the degradation of images which brings additional challenges for recognition. In the future, combining image enhancement algorithms to improve recognition accuracy would provide a promising research direction.

## Data availability statement

The raw data supporting the conclusions of this article will be made available by the authors, without undue reservation.

## Author contributions

RZ, LZ, and YS contributed to the conception and design of the study. RZ and QY organized the database. RZ performed the statistical analysis and wrote the first draft of the manuscript. RZ and GB wrote sections of the manuscript. LZ revised the article. All authors contributed to manuscript revision, read, and approved the submitted version.
